# The Growth of the Nursing Profession: VI. Opportunities of Study for the Graduate Nurse

**Published:** 1918-04-06

**Authors:** 


### THE MATRONS' AND SISTERS' DEPARTMENT.

### THE GROWTH OF THE NURSING PROFESSION.

### VI. Opportunities of Study for the Graduate Nurse.

People frequently ask why so much money is needed for the endowment of the College. If they would stop and think for a few minutes they would realise how extremely limited are the means remaining at the disposal of most hospitals when the interests of the patients have been adequately provided for. It is to the credit of the hospitals that in many cases large sums of money have been spent upon the nurses' homes and upon aids to training. In the case of the largest and wealthiest London and provincial hospitals it is quite within their power—thanks to the generous support which they receive from their public—to bring their nurses' homes up to a standard which shall satisfy modern requirements, and to give their nurses such assistance in training as shall make their theoretical work a matter of eager interest instead of an incessant nightmare. But the financial resources of the majority of hospitals are not equal to such a strain, and one part of the work of the College should be so to assist them in this direction that they shall be able to offer all the advantages now considered so essential in the training of the nurse. Nurses' brains are not always at their best or brightest either in the lecture hour or during the time which is at their disposal for study, and they are often obliged to waste a great many of the precious moments in the endeavour to grasp points which could have been made quite clear with the help of a really good model or some practical demonstration, or elucidated with the aid of a well-written and illustrated text-book. The worries of the anatomy examination would fade into insignificance if, for instance, the nurse had easy access to a complete set of bones, and were able to examine for herself models or specimens of the softer parts of the human organism. Every hospital should be equipped with these and other such-like aids to training, and especially should there be a thoroughly up-to-date library of well-chosen practical text-books, with some more advanced and specialised volumes for those particularly interested in any one branch or aspect of their work. The antiquated and dry-as-dust tomes bequeathed in past ages, which form the nurses library in too many institutions, are worse than useless. The unfortunate probationer gloomily contemplates their uninviting backs, and wonders despairingly which of them can possibly be of any use to her. And, indeed, for all the difference there is between them she might as well "toss up."

Then, again, the drawback to many small hospitals is that there is only one doctor to give all the lectures, and however excellent he may be he cannot be a specialist in everything, nor altogether avoid the tendency to impress his own ideas and opinions on his hearers. It is not a good thing for a nurse to have worked for one surgeon or one physician only, or to be under the impression that his is the only point of view. The provision of travelling lecturers and special courses of lectures, enabling the nurses to hear other points of view and get a wider grasp of their subjects, is a benefit which will be keenly appreciated in the smaller hospitals.

The nursing profession is probably the only one which offers no advantages to its members in the direction of post-graduate study, and one of the most attractive features of the College will be the provision of such opportunities. At the present time, when a nurse has gained her certificate, she takes up work at once and seeks no further opportunities for study. How many nurses join a co-operative or ordinary nursing institute at the completion of their training, and spend ten or twenty years in the same locality attached to the same institution? Their holidays are brief, their salary is not princely, and they cannot afford to travel abroad or to pay for further specialised training. In an adequately endowed College of Nursing the prospect opening before the nurse will be much more varied and attractive. If keen on study and eager for further knowledge she will make up her mind to gain a travelling scholarship, which will enable her either to take up some definite course of study abroad or to travel with the object of gaining knowledge as to how the nursing profession is equipped and how the nursing is done in other countries. Should she think that organisation and administration are her *forte*, special courses will be open to her which will soon discover for her whether she is right or no. Has she an inborn love of teaching and the gift of imparting her knowledge to others? Here there will be special opportunities for her, the chance of developing her gift to the utmost, and of obtaining a post where she will be able to exercise it unfettered by the demands of a hundred other just as important aspects of work. Is she impressed with the urgency of infant welfare, or do the aged appeal more particularly to her? For every branch of the nurse's work there will be special courses and facilities, so that she will come well equipped to her chosen task, and will avoid on the one hand the mistakes which must often be made by the best when they enter upon new work of which they have had no manner of experience, and, on the other, the disappointment that comes to the woman who imagines she will like a particular branch of work, and then finds that she has not the qualities requisite for success therein. Needless to say, the public as well as the nurses will benefit incalculably, and it is the realisation of results of this nature which will bring home to the public not only the excellent principle of the College of Nursing, but also its tremendous possibilities as a power for good in the nation and the need for an endowment on a generous scale.

☛ Previous articles appeared Jan. 19, 26; Feb. 9, 23; and March 16, p. 507.

